# 1870. Changes of Tuberculosis Incidence and Mortality after COVID-19 Pandemic: A Interrupted Time-series Analysis

**DOI:** 10.1093/ofid/ofad500.1698

**Published:** 2023-11-27

**Authors:** Jungju Lee, Byung Chul Chun

**Affiliations:** Korea University College of Medicine, Seoul, Seoul-t'ukpyolsi, Republic of Korea; Korea University College of Medicine, Seoul, Seoul-t'ukpyolsi, Republic of Korea

## Abstract

**Background:**

The objective of this study is to investigate how the incidence and mortality levels and trends of tuberculosis changed before and after the COVID-19 pandemic in Korea.

**Methods:**

To estimate the incidence rate of tuberculosis in Korea, we calculated the number of reported cases per year from 2015 to 2021 and divided it by the corresponding mid-year population. Data on reported cases were obtained from the Korea Centers for Disease Control and Prevention, which mandates the reporting of tuberculosis. Descriptive epidemiology was used to analyze monthly incidence and mortality rates, and the Durbin-Watson test was employed to assess seasonality and time-series autocorrelation. Interrupted time series analysis (ITS) using ARIMA models was performed on both the incidence and mortality rates, with the starting point of the COVID-19 pandemic set at February 2020, when the first cases emerged in the community. The tuberculosis mortality was analyzed separately for two age groups: those aged 65 and above, and those under 65.

**Results:**

During the study period, a total of 204,190 tuberculosis cases were reported, with 12,407 deaths identified. The ITS results showed that the incidence had a significant decreasing trend before the COVID-19 pandemic (b=-0.024, p< 0.01) and a significant decrease in the level of incidence after the pandemic (b=-0.72, p< 0.01). The decreasing trend of incidence was not statistically significant after the pandemic. The mortality had a continuous decreasing trend before the pandemic (b=-0.002, p< 0.01), and the level of mortality significantly decreased after the pandemic (b=-0.029, p< 0.01). However, the trend of mortality increased significantly after the pandemic (b=0.003, p< 0.01). When analyzed by age group, the increase in mortality rate was significantly evident in those aged 65 and above (b=0.016, p< 0.01), while it was not significant in those under 65 (b=0.002, p=7.08).

**Figure:**
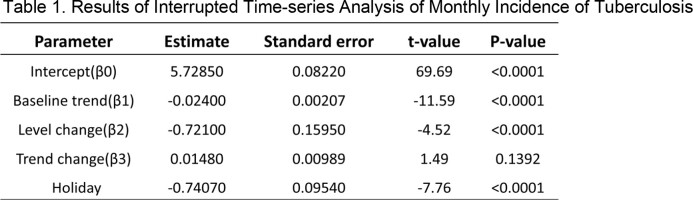

**Figure:**
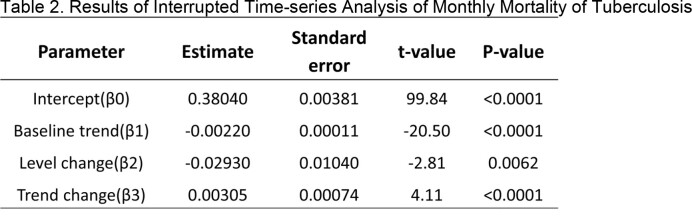

**Conclusion:**

After the COVID-19 pandemic, there was a decrease in the incidence of tuberculosis, which resulted in a decrease in the tuberculosis mortality rate. However, in contrast to the pre-pandemic period, the tuberculosis mortality rate has shown a significant increasing trend since the pandemic, indicating collateral damage to tuberculosis patients due to the COVID-19 pandemic.

**Disclosures:**

**All Authors**: No reported disclosures

